# Evaluation of an Artificial Intelligence-Assisted Metaphase Finder for Clinical Cytogenetic Application

**DOI:** 10.3390/genes17101295

**Published:** 2026-10-16

**Authors:** Bilinda J. Dawson, Christy A. Prongay, Tori B. Strapp, Lynne S. Rosenblum

**Affiliations:** 1 Cytogenetics Laboratory, Atrium Health Wake Forest Baptist Medical Center, Winston-Salem, NC 27157, USA; bilinda.dawson@advocatehealth.org (B.J.D.); christy.prongay@advocatehealth.org (C.A.P.); tori.strapp@advocatehealth.org (T.B.S.); 2 Department of Pathology, Wake Forest University School of Medicine, Winston-Salem, NC 27157, USA

**Keywords:** artificial intelligence (AI), clinical cytogenetics, laboratory genetics, metaphase finder classifier, metaphase detection

## Abstract

Background/Objectives: Clinical cytogenetics has benefited from artificial intelligence (AI) integration into digital karyotyping systems for segmentation and classification of chromosomes. Metaphase image identification and capture utilize traditional metaphase finder classifiers whose specificity can be improved, especially where lowered detection thresholds for low-mitotic-index specimens result in higher debris selection. Use of AI to reliably detect metaphases at 10× magnification may resolve this issue. This study compares a new AI-based metaphase finder algorithm to the conventional non-AI classifier. Methods: G-banded slides from 105 normal and abnormal peripheral blood (PB) and bone marrow (BM) specimens were scanned at 10× magnification and images were captured at 100× using the HiBand system (Applied Spectral Imaging), once with the automatic metaphase finder and again using the AI-assisted algorithm. The number of real metaphases versus debris, for up to 100 captured images per slide, and quality metrics were compared using both applications. Results: True metaphase detection in BM specimens averaged 77.3 ± 21.6 versus 82.5 ± 19.9 (6.3% increase) for normal cases and 72.1 ± 18.5 versus 89.7 ± 14.2 (14.6% increase) for abnormal cases comparing the non-AI and AI classifiers, respectively. There was no difference for normal PB cases (97.6 ± 1.5 versus 97.1 ± 2.9) and a 2.6% decrease for abnormal PB cases (97.9 ± 2.2 versus 95.4 ± 4.2) between the two classifiers. All cases demonstrated comparable image quality. Conclusions: This study demonstrates that the AI-assisted metaphase finder improves true metaphase capture compared to the non-AI classifier for BM while maintaining high fidelity for PB specimens. These findings contribute to the expanding literature on integration of AI in clinical cytogenetics.

## 1. Introduction

Cytogenetic analysis has been used in the clinical laboratory since the 1970s to identify numerical and structural chromosome abnormalities in patients with constitutional genetic disorders and cancers [1,2]. Despite the advances conferred to the field by molecular diagnostic techniques, chromosome analysis remains an integral component of genetic testing [3,4,5,6,7,8], providing structural information and single-cell granularity not possible with methods reliant upon extracted DNA.

Early, classic cytogenetic analysis was a labor-intensive manual process involving cell culture, preparation of slides, scanning G-banded slides under a microscope to identify metaphase spreads, photographing and printing images, cutting out individual chromosomes, and arranging the chromosomes in a karyogram on a paper template for analysis. Upgrades in the 1990s provided automation of slide scanning, digital image capture, and semi-automated karyotyping processes [9,10]. Subsequent incremental improvements in the next decades enhanced these functions [11,12] but lacked optimal specificity and accuracy. In the past few years, introduction of artificial intelligence (AI)-based algorithms for clinical karyotyping has significantly enhanced the accuracy and efficiency of the karyotyping process [13,14,15,16,17,18].

Despite integration of AI for karyotype generation, the preceding workflow step of scanning the slide to identify metaphase candidates for image capture continued to utilize traditional automated metaphase finder classifiers. Slide scanning employs a similar process whether using completely manual methods, automated software, AI-based algorithms, or a combination thereof. Briefly, there is an initial scan at low magnification to identify cell clusters and metaphase candidates. Next, a second scan is performed to select analyzable metaphases, which are then captured at higher magnification as photographic images for subsequent analysis. Accurate identification of analyzable metaphase cells is thus critical to the entire karyotyping process [19].

Conventional automated metaphase finder classifiers require laboratory-specific adjustments of default settings, but with customization can provide high specificity for ideal specimens, such as peripheral blood (PB) with robust cellularity, high mitotic index, and high band resolution. However, specimens with a lower mitotic index and poorer chromosome quality require lowering detection thresholds to identify all possible metaphases, resulting in selection of more debris images. In such cases, the specificity for detection of true metaphases versus debris can be improved. It is therefore necessary to develop more robust metaphase finder algorithms, which are applicable to a variety of specimen types and slide preparation methods, to reduce the need to perform custom adjustments.

AI-based metaphase finder classifiers have the potential to address this requirement, and several such products have been reported in the research space or as laboratory-developed tests at individual institutions [19,20,21,22,23,24]. Commercial applications for clinical use have only become available very recently [25,26] and, while initial reports are promising [27,28], their utility has not been fully assessed. It is critically important to vet any new AI-based software which is integral in presenting data utilized for clinical review and patient diagnosis. In this paper, we report on the evaluation of a new AI-based metaphase finder algorithm compared to the conventional non-AI classifier for 105 PB and bone marrow (BM) specimens. To reflect real-world implementation in the clinical laboratory, and to evaluate the inherent integrity of the AI-based software, this comparison was performed with the custom optimized non-AI classifier currently used in our clinical laboratory versus the AI-based classifier with the manufacturer’s default settings.

## 2. Materials and Methods

### 2.1. Specimen Selection and Preparation

A total of 105 specimens were selected from completed clinical cytogenetic cases tested in the Cytogenetics Laboratory, Atrium Health Wake Forest Baptist Medical Center, and comprised 25 PB with normal karyotypes, 25 PB with abnormal karyotypes, 25 BM with normal karyotypes, and 30 BM with abnormal karyotypes. PB specimens were cultured for 72 h in Chang medium MF (Fujifilm Irvine Scientific, Santa Ana, CA, USA) and BM specimens were cultured in Chang marrow medium overnight or for 24 or 48 h, with colcemid or pokeweed antigen added if indicated by clinical diagnosis. One slide was prepared manually for each case and G-banded with trypsin-Giemsa (GTG) staining. Slides were baked for 30–45 min at 90 °C. Specimen setup and harvest were performed by standard techniques [29,30]. The average band resolution ranged from 350 to 650 bands per haploid set. After pertinent technical information was collected, these specimens were de-identified for use in this study.

### 2.2. Slide Scanning

The G-banded slides were scanned overnight twice, on a 99+-slide tray loader, first using the HiBand system (Applied Spectral Imaging, Carlsbad, CA, USA) version 8.4.1 (v8.4.1) with automated (non-AI) metaphase detection and again with HiBand v8.4.2 and its AI-based metaphase detection software. Briefly, a 10× objective was used to scan slides and identify metaphase cells and a 100× objective was used to capture a maximum of 100 high-definition images using a Zeiss Z2 microscope (Carl Zeiss Microscopy, LLC, White Plains, NY, USA) with a black and white, five-megapixel camera. For v8.4.1, scanning criteria had been configured and optimized for our clinical laboratory use. The AI-assisted v8.4.2 was employed as provided by the manufacturer, with no customization.

### 2.3. Comparison of Performance

**Figure 1:**
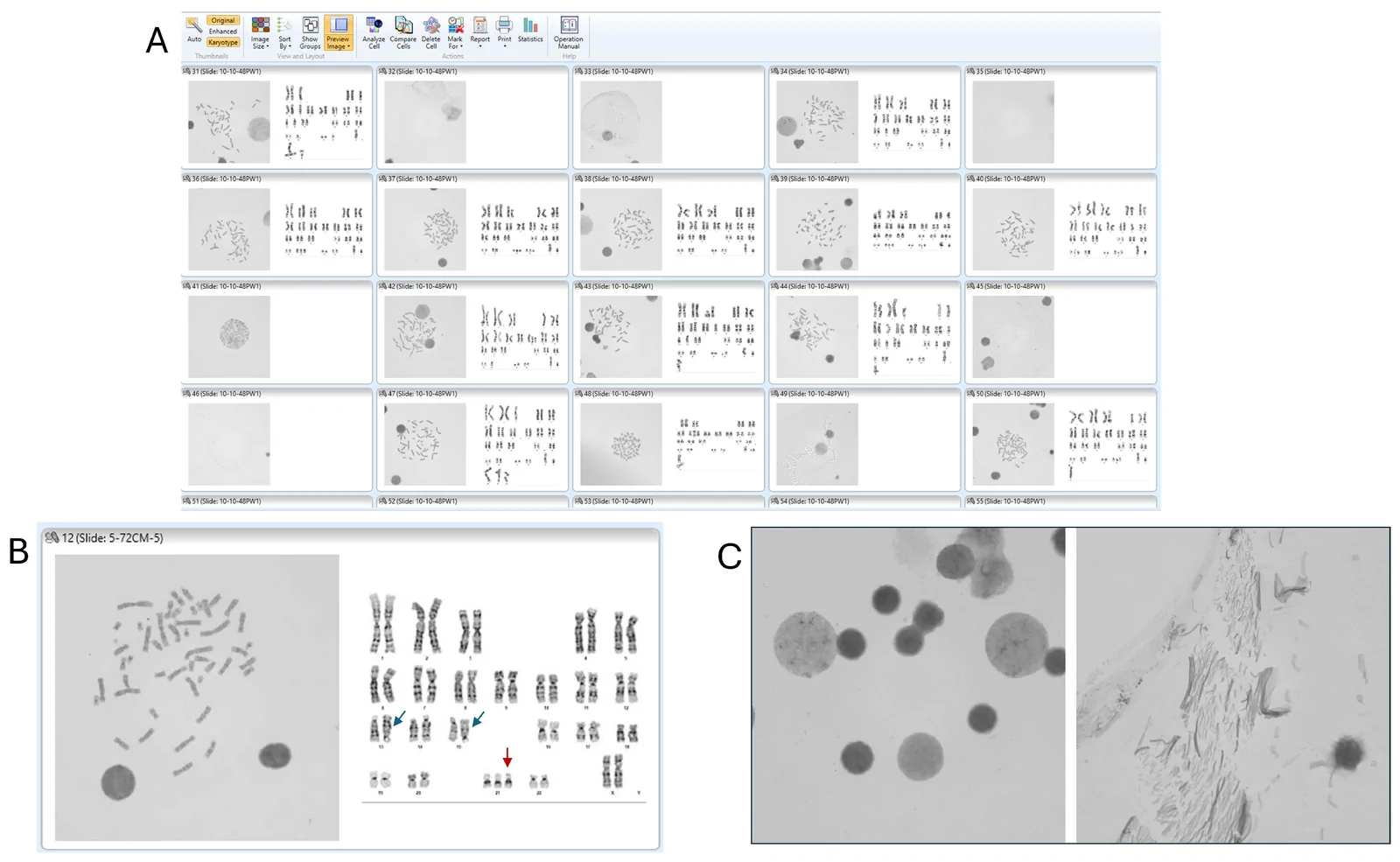
Representative visuals used to compare the performance of the HiBand version 8.4.1 automated metaphase finder classifier versus the HiBand version 8.4.2 AI-based classifier. (**A**) Excerpt of the low-definition image gallery used to view and evaluate the number of true metaphases versus debris images captured. Case BMNL10 is shown. (**B**) Example of an uncorrected metaphase-karyogram pair generated by the AI-karyotyping algorithm, taken from specimen PBAB5. Note trisomy 21 (red arrow) and the upside-down chromosomes 13 and 15 (blue arrows). (**C**) Examples of non-metaphase images of interphase nuclei (**left**) and Giemsa stain residue (**right**) captured by the automated version of the metaphase finder classifier.

The results of the two scans were assessed by viewing the images in the ASI image gallery (Figure 1A) and recording associated numeric data for each case. A comparison of the two applications was performed based on the following criteria for each case: total number of images captured and number of true metaphases identified versus debris. The sorting was binary. An image was considered a true metaphase regardless of chromosome quality or spread, or whether it had been AI-karyotyped (Figure 1B). Any image which contained overlapping or multiple separated metaphases was counted as a true metaphase. An image containing only other content, such as Giemsa stain residue or interphase nuclei, was counted as debris (Figure 1C).

Granular data and overall performance for each specimen category were tabulated and summarized. Average, standard deviation, range, and median were compared for each dataset. Statistical significance was assessed using a Wilcoxon Signed-Rank test for paired samples, specifically when comparing each specimen type scanned with the two versions of the software. A Bonferroni correction for multiple comparisons was also calculated. A Mann–Whitney U test for independent samples was used to compare the two PB sets and the two BM sets scanned with the AI algorithm.

As a quality metric, the number of metaphases karyotyped by the AI-assisted algorithm for each case was recorded and compared for both scans. Concordance with the clinical reported karyotype was confirmed by manual image review of the first 20 karyograms from each scan. Estimation of average band resolution and concordance with the clinically reported value was determined by the reviewing technologist and served as an additional proxy for quality assessment.

## 3. Results

A total of 105 slides comprised 25 normal and 25 abnormal PB specimens, and 25 normal and 30 abnormal BM specimens were included in this evaluation of an AI-based metaphase finder classifier, using the manufacturer’s default settings, compared to an optimized automated classifier. For all four datasets, assessment of the first 20 AI-karyograms per slide demonstrated the karyotype, band resolution, and overall image quality, being concordant for all cases when comparing the two classifier versions, and there was no evidence of AI-generated false information. Since the scoring of true metaphases versus debris was binary, the results are complementary for each dataset. For simplicity of data presentation, we primarily address findings for true metaphases, which are the clinically meaningful images, but the complete datasets are shown in Supplementary Tables S1–S4.

### 3.1. Peripheral Blood Specimens—Captured Images and True Metaphases

**Figure 2:**
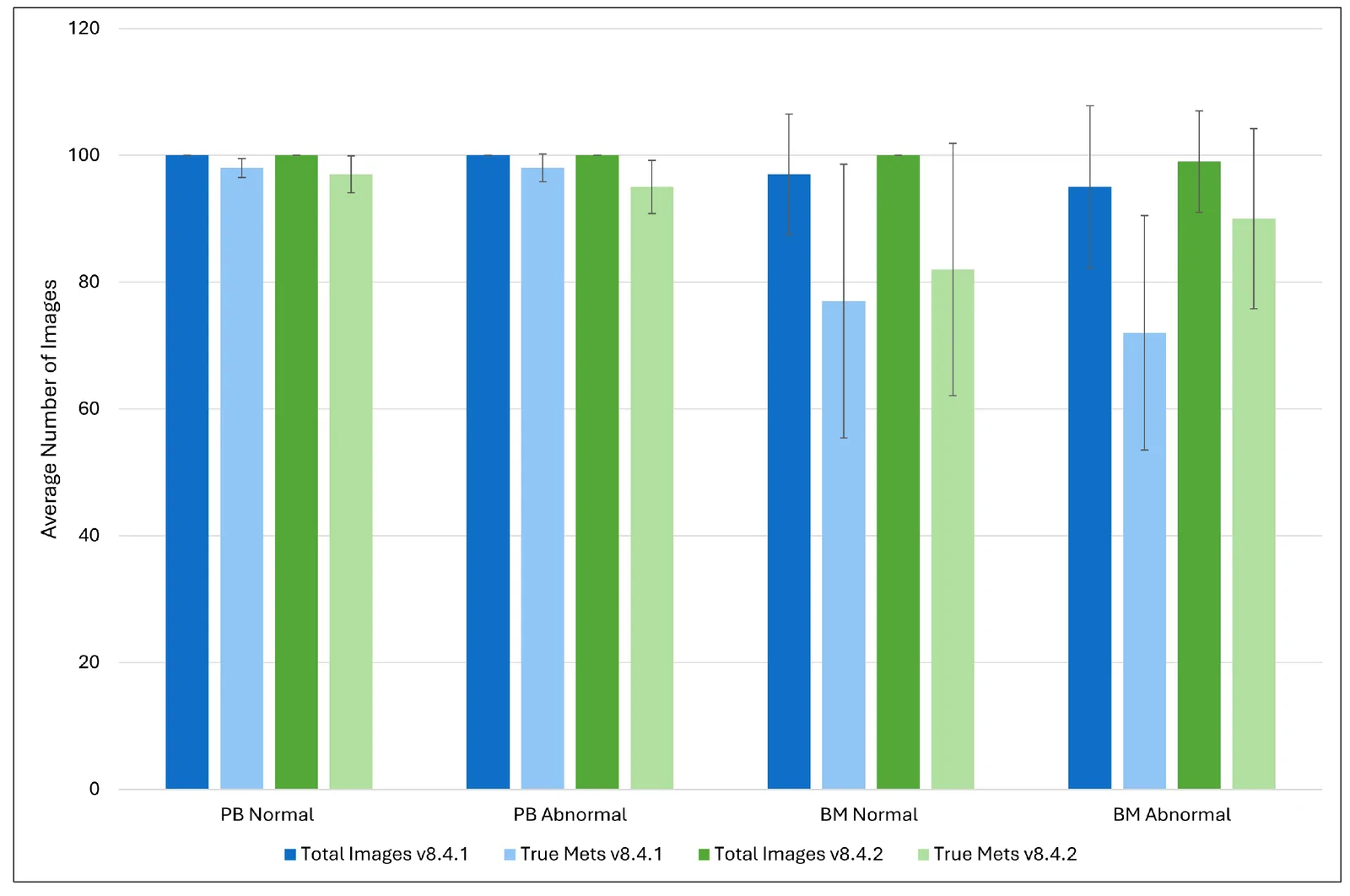
Box and whisker plot showing the average number of total images captured (darker color) and corresponding true metaphases (mets) identified (lighter color), comparing the automatic v8.4.1 (blue) versus AI-based v8.4.2 (green) metaphase finder classifier algorithms. The results are grouped by specimen type: peripheral blood (PB) or bone marrow (BM) and normal or abnormal karyotype. Underlying data are provided in Supplementary Tables S1–S4.

For PB specimens, a full set of 100 images was captured for each normal and abnormal case with both the automated v8.4.1 and AI-assisted v8.4.2 algorithms. The v8.4.1 scan of the 25 normal cases identified an average of 97.6 ± 1.5 true metaphases (range 95–100, median 98) compared with an average of 97.1 ± 2.9 true metaphases (range 88–100, median 97) (Table S1). There was no statistical difference between average number of metaphases detected with the two versions. The v8.4.1 scan of the 25 abnormal cases identified an average of 97.9 ± 2.2 true metaphases (range 90–100, median 98) compared with an average of 95.4 ± 4.2 true metaphases (range 86–100, median 97) using v8.4.2 (Table S2), demonstrating 2.6% fewer metaphases detected (*p* = 0.01) (Table 1, Figure 2). There was no statistical difference between the average number of metaphases detected for normal (97.1 ± 2.9) versus abnormal (95.4 ± 4.2) PB specimens with v8.4.2 scanning.

### 3.2. AI-Karyotyped Peripheral Blood Specimens

Regarding AI-assisted karyotyping of the captured metaphase images, the normal PB specimens scanned with v8.4.1 had an average of 84.7 ± 5.7 (87% of true metaphases) AI-karyotyped metaphases versus a lower 63.6 ± 10.2 (66%) with v8.4.2, which was significant (*p* < 0.0001). The v8.4.1 scan of abnormal PB had an average of 78.0 ± 12.7 (80%) images AI-karyotyped compared to 64.1 ± 15.6 (67%) AI-karyotyped after scanning with v8.4.2, which was also significant (*p* = 0.003) (Table 2).

**Figure 3:**
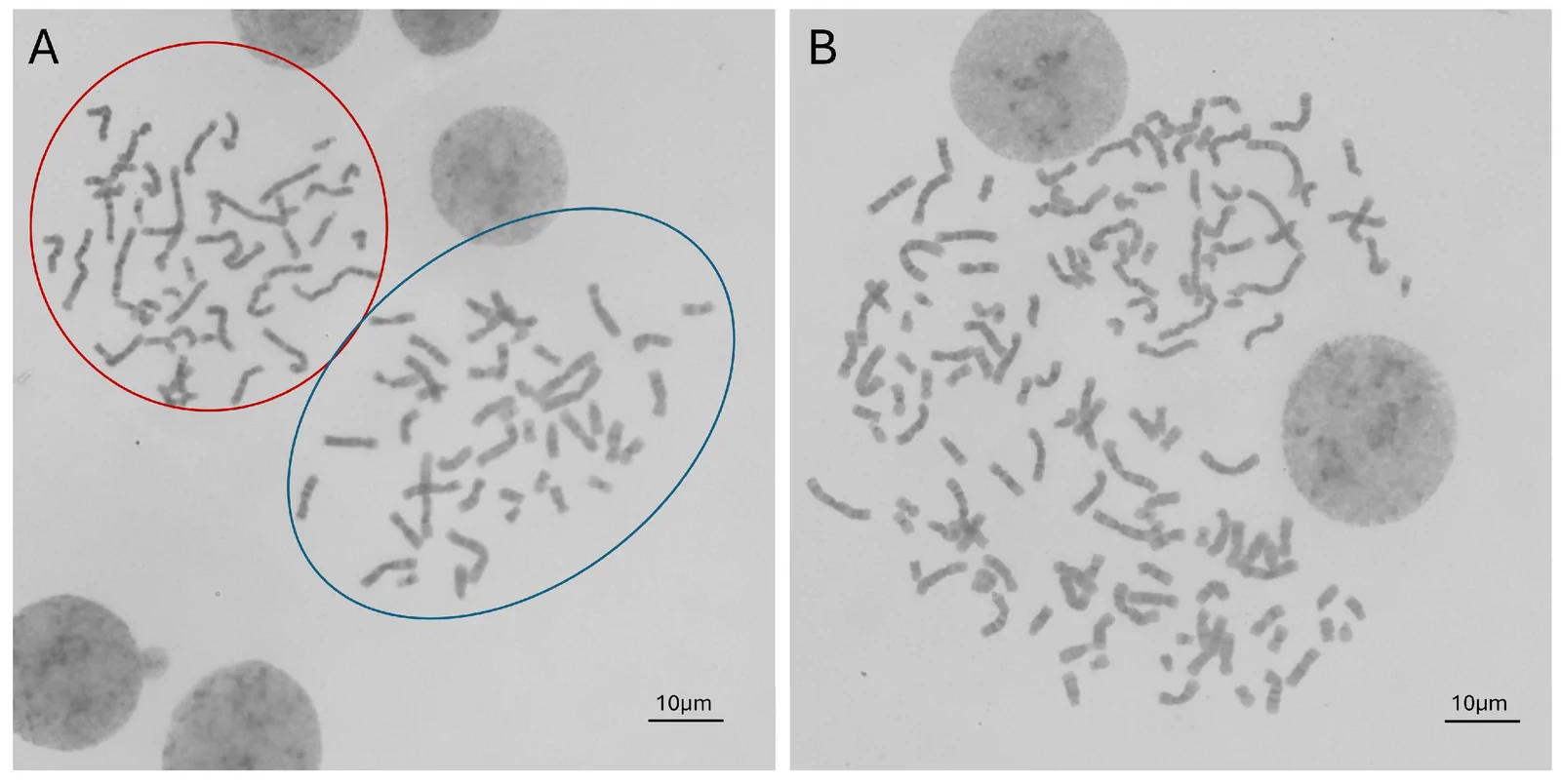
Representative true metaphase images from peripheral blood specimens which were captured by the AI-based v8.4.2 metaphase finder classifier and not karyotyped by the AI-assisted karyotyping algorithm. Multiple metaphases were captured within each image. (**A**) Image containing two distinct metaphases, shown circled in blue and red. (**B**) Image containing three overlapping metaphases. Images were captured at 100× magnification; scale bar represents approximately 10 µm.

Of note, a large proportion of the non-karyotyped true metaphases contained multiple metaphases in the individual captured images using v8.4.2, for both normal and abnormal cases (Figure 3, Tables S1 and S2).

### 3.3. Bone Marrow Specimens—Captured Images

For BM specimens, a full set of 100 images was captured for most but not all cases, due to the low mitotic index of some specimens. Three of 25 normal karyotype cases (BMNL3, BMNL18, BMNL24) achieved 90, 55, and 84 images respectively with v8.4.1 scanning, but 100 images were obtained for all 25 cases with v8.4.2 scanning (Table S3). Among the 30 abnormal cases scanned with v8.4.1, six cases had fewer than 100 images captured (cases BMAB10, BMAB15, BMAB16, BMAB20, BMAB22, and BMAB123, with 90, 62, 81, 42, 95, and 91 images respectively), and when scanned with v8.4.2, all cases obtained 100 images except BMAB20, which had 56 images (Table S4).

### 3.4. Bone Marrow Specimens—True Metaphases

The v8.4.1 scan of the 25 normal BM cases identified an average of 77.3 ± 21.6 true metaphases (range 6–98, median 82) compared with an average of 82.5 ± 19.9 true metaphases (range 32–100, median 89) using v8.4.2 (Table S3), demonstrating 6.3% more metaphases detected with v8.4.2 compared to v8.4.1 (*p* = 0.04). The v8.4.1 scan of the 30 abnormal cases identified an average of 72.1 ± 18.5 true metaphases (range 7–95, median 76) compared to the v8.4.2 scan with an average of 89.7 ± 14.2 true metaphases (range 24–99, median 93) (Table S4). This resulted in 14.6% more metaphases detected with v8.4.2 compared to v8.4.1 (*p* < 0.0001) (Table 1, Figure 2). There was no statistical difference between the average number of metaphases detected for normal (82.5 ± 19.9) versus abnormal (89.7 ± 14.2) BM specimens with v8.4.2.

### 3.5. AI-Karyotyped Bone Marrow Specimens

The BM specimens had more AI-karyotyped metaphase images and a higher percentage of total true metaphases AI-karyotyped with v8.4.2 than for v8.4.1. Normal specimens scanned with v8.4.1 had an average of 67.1 ± 20.8 (87%) AI-karyotyped versus 73.4 ± 21.7 (89%) with v8.4.2. The v8.4.1 scan of abnormal BM specimens had an average of 57.8 ± 17.4 (80%) images AI-karyotyped compared to 72.3 ± 22.8 (81%) AI-karyotyped after scanning with v8.4.2 (Tables S3 and S4). The BM specimens had more AI-karyotyped metaphases images for both normal (*p* = 0.04) and abnormal (*p* < 0.001) cases when scanned with v8.4.2 compared to v8.4.1 (Table 2).

## 4. Discussion

The integration of AI into clinical laboratory processes such as cytogenetic analysis is important because it can automate repetitive and labor-intensive processes with enhanced efficiency and accuracy compared to non-AI tools [12,17,31]. However, prior to implementation of any new AI-based product, the reliability and limitations of the algorithm should be evaluated and understood in the context in which it will be used [18].

The goal of this study was to determine the effectiveness of an AI-trained metaphase finder in the clinical setting by integrating the “out-of-the-box” software into the workflow and practices currently in operation in a clinical cytogenetics laboratory. It was also important to evaluate the verity of the output, notwithstanding this AI-based algorithm is iterative, not generative. Unlike AI-assisted karyotyping where a karyogram is available for review and correction, the metaphase finder classifier shows only the images chosen and thus creates a blind spot for quality assurance.

This evaluation of the ASI HiBand v8.4.2 AI-assisted metaphase finder utilized with its default settings demonstrated that it improves true metaphase versus debris capture when compared to the customized non-AI classifier for BM while maintaining high fidelity for PB specimens (Table 1). All cases were concordant for karyotype, band resolution, and image quality when comparing the two scanning algorithms, assuring that the AI-based metaphase finder had no impact on photographic quality. However, differences were observed when comparing metaphase finder classifier functions for different specimen types, namely PB and BM.

The optimized automated metaphase finder classifier v8.4.1 exhibited high accuracy for identification of true metaphases in PB cases with normal and abnormal karyotypes and served as a baseline for this comparison. Similar accuracy was achieved with the AI-based classifier v8.4.2 for normal cases, but for abnormal PB, the average number of true metaphases identified was 2.6% fewer using the default AI classifier v8.4.2 (95.4 ± 4.2 versus 97.9 ± 2.2 with v8.4.1). Minor customization of the AI program settings might improve this outcome, but in the context of clinical utility, adjustments may not be necessary, given that in all cases, at least 86 true metaphases were captured from the slide. Per technical guidelines [30,32], case completion requires at least 20 metaphases from two different cultures, and thus two slides (one from each culture) would exceed that requirement.

There was an unexpected finding with the PB cases in which many true metaphase images were not karyotyped by the AI-assisted algorithm when scanned with v8.4.2. Although AI-assisted karyotyping was not evaluated as part of this study, it was used as a tool to assess quality metrics, and therefore this finding was concerning. Investigation revealed that these non-karyotyped images contained two or more metaphases (Figure 3, Tables S1 and S2) and thus did not meet numeric criteria for the AI-assisted karyotyping algorithm. This issue was generally not observed for the BM specimens, where a single exception occurred in each set (specimens BMNL12 and BMAB19). To better understand this phenomenon, in silico assessment of select PB cases with high numbers of multiple-metaphase non-karyotyped images was performed and demonstrated a single adjustment, reducing the image box size from 180 to 100 pixels and reducing the number of non-karyotyped images to levels similar to those of v8.4.1. This suggests that the issue was an artifact of the default parameters of v8.4.2, which may benefit from minor adjustments depending on specimen type and clinical laboratory practice. This observation also illustrates the need for a human component in the optimal use of AI-based tools.

The important benefit conferred by the AI metaphase finder v8.4.2 was for BM specimens, where reliable metaphase detection is required to meet the requirement that analysis includes metaphase spreads representing a range of both good and poor morphology [33,34]. This is critical to confidently identify or rule out clonal chromosome abnormalities which impact clinical diagnosis and patient care [35]. For these specimens, improvements were observed with the AI-based v8.4.2 scanning algorithm for both the number of images captured and number of true metaphases identified (Figure 2). Of note, the 6.3% increase in overall true metaphase detection for normal BM specimens was not statistically significant when a Bonferroni correction was applied (Table 1). However, each data point represents an individual patient for whom the cytogenetic result impacts clinical care. Briefly, of the three normal BM cases with fewer than 100 images captured by v8.4.1, all were complete, and of the six abnormal BM cases with fewer than 100 images with v8.4.1, five were complete with v8.4.2. Additionally, v8.4.2 presented more true metaphases for 17 of 25 normal cases and 29 of 30 abnormal cases (Tables S3 and S4). This demonstrates that for specimens with suboptimal mitotic index and/or metaphase quality, the AI-based classifier provides improved identification of true metaphases.

Of note, for the abnormal BM specimens, the first 25 cases were generally matched to the complexity of the abnormal PB specimens, which served as our high-fidelity baseline. Five more-complex hyperdiploid specimens were added to challenge the AI-based v8.4.2 algorithm, and the number of true metaphases identified was within the observed range for the first 25 specimens with both algorithms. Therefore, there was no discernable pattern in the improved metaphase detection with respect to the karyotype of the abnormal cases (Table S4). Extremely abnormal BM cases, such as those with near-tetraploidy or more than three clonal cell lines, were not included in the dataset; covering the full spectrum of complex abnormal BM karyotypes was beyond the scope of this project. However, the average number of true metaphases identified improved with v8.4.2 compared to v8.4.1 for both normal and abnormal BM cases, with 6.3% and 14.6% more metaphases detected, respectively. These findings combine to suggest that cytogenetic complexity and metaphase morphology do not significantly influence the effectiveness of the AI-based metaphase finder classifier.

Employment of AI-based software for metaphase detection and capture is reported to decrease scanning time relative to non-AI processes for several different algorithms [19,20,25]. In this study, it was not possible to compare on-instrument scanning time between the two algorithms, due to our system set-up. In our laboratory, the slide scanning process is dependent on our shared medical center computer servers, where the unpredictable network traffic may create bottlenecks and impact scan time. A laboratory with a dedicated server could likely investigate this interesting metric, although the workflow impact may be minimal if scanning is performed overnight.

Overall, the performance of the AI-trained metaphase finder classifier provided a high level of confidence in the algorithm output, which in turn has the potential to provide improvement in clinical laboratory workflow and efficiency for metaphase assessment. Confidence that all available metaphases, up to a pre-set limit, have been identified and captured assures the technologist that there would be limited utility in manually scanning the slide to identify missed metaphases. The corollary is when an insufficient number of metaphases are identified, the technologist knows their time will be well spent preparing and scanning the additional slides necessary to complete the case. In terms of the images presented in the gallery, the high proportion of true metaphases versus debris reduces the need to remove debris images or mark and sort usable images to analyze the case. The improvements allow technologists to concentrate their focus on more challenging tasks for case review and analysis, leading to greater job satisfaction. These factors are particularly important at a time when there are growing constraints in the genomic laboratory workforce [36].

## 5. Conclusions

In summary, this single-center study evaluated the early release version of the ASI HiBand v8.4.2 AI-based metaphase finder algorithm to assess the frontline user utility of the product with its default settings. The comparison was between an optimized non-AI version and a default AI-based version of the software, not default versions of both. This choice was intentional and provided insight into the effect of a direct upgrade, without time-consuming customization, of a system currently in use in a clinical laboratory. Our results demonstrate the efficacy conferred by the AI algorithm in detection and image capture of true metaphases versus debris for PB specimens and improved performance for BM specimens, particularly those with low mitotic index. The study also illustrates the critical role of human review in optimizing AI-based tools and assessing their output when implementing in the clinical laboratory. Additional studies evaluating this emerging technology across laboratories and commercially available products will be needed as integration of AI becomes standard practice in clinical cytogenetics.

## Figures and Tables

**Table 1:** Comparison of the average number of true metaphases detected among total images captured with the automatic (v8.4.1) versus the AI-based (v8.4.2) metaphase finder classifier algorithms for the four specimen types. Underlying data are provided in Supplementary Tables S1–S4.

Specimen Type	v8.4.1	v8.4.2	*p*-Value α = 0.05	Difference in Ave. Number Metaphases Detected
PB Normal	97.6 ± 1.5	97.1 ± 2.9	0.42	No difference
PB Abnormal	97.9 ± 2.2	95.4 ± 4.2	0.01	2.6% less with v8.4.2
BM Normal	77.3 ± 21.6	82.5 ± 19.9	0.04 *	6.3% more with v8.4.2
BM Abnormal	72.1 ± 18.5	89.7 ± 14.2	<0.0001	14.6% more with v8.4.2

AI, artificial intelligence; v8.4.1, automatic metaphase finder; v8.4.2, AI-based metaphase finder; PB, peripheral blood; BM, bone marrow; *, not significant after Bonferroni correction where α = 0.0125.

**Table 2:** Comparison of the average number of metaphases AI-karyotyped among the metaphases captured with the automatic (v8.4.1) versus the AI-based (v8.4.2) metaphase finder classifier algorithms for the four specimen types. Underlying data are provided in Supplementary Tables S1–S4.

Specimen Type	v8.4.1	v8.4.2	*p*-Value α = 0.05	Difference in Ave Number Metaphases Karyotyped
PB Normal	84.7 ± 5.7	63.6 ± 10.2	<0.0001	33.3% less with v8.4.2
PB Abnormal	78.0 ± 12.7	64.1 ± 15.6	0.003	21.8% less with v8.4.2
BM Normal	67.1 ± 20.8	73.4 ± 21.7	0.04 *	8.6% more with v8.4.2
BM Abnormal	57.8 ± 17.4	72.3 ± 22.8	<0.0001	20.0% more with v8.4.2

AI, artificial intelligence; v8.4.1, automatic metaphase finder; v8.4.2, AI-based metaphase finder; PB, peripheral blood; BM, bone marrow; *, not significant after Bonferroni correction where α = 0.0125.

## Data Availability

The original contributions presented in this study are included in the article/Supplementary Material. Further inquiries can be directed to the corresponding author.

## Supplementary Materials

The following supporting information can be downloaded at https://www.mdpi.com/article/10.3390/genes17101295/s1, Table S1: Comparative data and analysis of peripheral blood specimens with normal karyotypes scanned with the automated and AI-based metaphase finders; Table S2: Comparative data and analysis of peripheral blood specimens with abnormal karyotypes scanned with the automated and AI-based metaphase finders; Table S3: Comparative data and analysis of bone marrow specimens with normal karyotypes scanned with the automated and AI-based metaphase finders; Table S4: Comparative data and analysis of bone marrow specimens with abnormal karyotypes scanned with the automated and AI-based metaphase finders.
